# A randomized controlled trial to evaluate and assess the effect of comprehensive pre-end stage kidney disease education on home dialysis use in veterans, rationale and design

**DOI:** 10.1186/s12882-022-02740-8

**Published:** 2022-03-30

**Authors:** Ashutosh M. Shukla, Jennifer Hale-Gallardo, Tatiana Orozco, Ivette Freytes, Zachary Purvis, Sergio Romero, Huanguang Jia

**Affiliations:** 1 North Florida / South Georgia Veteran Healthcare System, Gainesville, FL USA; 2 Division of Nephrology, Hypertension and Transplantation, University of Florida, 1600 Archer Road, Gainesville, FL 32610 USA

**Keywords:** Chronic kidney disease, Renal insufficiency, Kidney failure, End stage kidney disease, Patient-centered care, Shared decision making, Health education, Quality of life, home Dialysis

## Abstract

**Background:**

Informed dialysis selection and greater home dialysis use are the two long-desired, underachieved targets of advanced chronic kidney disease (CKD) care in the US healthcare system. Observational institutional studies have shown that comprehensive pre-kidney failure, conventionally referred to as end stage kidney disease education (CPE) can improve both these outcomes. However, lack of validated protocols, well-controlled studies, and systemic models have limited wide-spread adoption of CPE in the US. We hypothesized that a universal CPE and patient-centered initiation of kidney replacement therapy can improve multiple clinical, patient-centered and health service outcomes in advanced CKD and kidney failure requiring dialysis therapy.

**Methods:**

Trial to Evaluate and Assess the effects of CPE on Home dialysis in Veterans (TEACH-VET) is a multi-method randomized controlled trial aimed to evaluate the effects of a system-based approach for providing CPE to all Veterans with advanced CKD across a regional healthcare System. The study will randomize 544 Veterans with non-dialysis stage 4 and 5 CKD in a 1:1 allocation stratified by their annual family income and the stage of CKD to an intervention (CPE) arm or control arm. Intervention arm will receive a two-phase CPE in an intent-to-teach manner. Control arm will receive usual clinical care supplemented by resources for the freely-available kidney disease information. Participants will be followed after intervention/control for the duration of the study or until 90-days post-kidney failure, whichever occurs earlier.

**Results:**

The primary outcome will assess the proportion of Veterans using home dialysis at 90-days post-kidney failure, and secondary outcomes will include post-intervention/control CKD knowledge, confidence in dialysis decision and home dialysis selection. Qualitative arm of the study will use semi-structured interviews to in-depth assess Veterans’ satisfaction with the intervention, preference for delivery, and barriers and facilitators to home dialysis selection and use. Several post-kidney failure clinical, patient-centered and health services outcomes will be assessed 90-days post-kidney failure as additional secondary outcomes.

**Conclusion:**

The results will provide evidence regarding the need and efficacy of a system-based, patient-centered approach towards universal CPE for all patients with advanced CKD. If successful, this may provide a blueprint for developing such programs across the similar healthcare infrastructures throughout the country.

**Trial registration:**

NCT04064086.

## Background

Progressive chronic kidney disease (CKD) and resultant kidney failure, conventionally referred to as ‘end stage kidney disease (ESKD)’ are huge public health burdens with high morbidity and mortality, poor health-related quality of life (HRQoL) and disproportionately high healthcare expenditure. Over 97% of incident kidney failure patients are managed by dialysis therapy [1]. Despite equivalent survivals and trends for better patient-reported and health services outcomes, use of various forms of home dialysis therapies remains low (~ 10%) among the US kidney failure population, [1, 2] and ~ 90% of incident and prevalent kidney failure requiring dialysis therapy are managed by in-center hemodialysis. It is estimated that doubling the current home dialysis rates would save over a billion Medicare dollars each year though, it is uncertain whether these cost savings merely represent the bias in patient selection existent for home dialysis [3]. Major stakeholders in kidney disease including providers, professional renal societies, patient advocacy groups, and payors such as Center for Medicare and Medicaid Services (CMS) and the Veterans Health Administration’s (VHA) National Kidney Program recommend increasing home dialysis utilization for the management of kidney failure requiring dialysis [4, 5].

Professional organizations recommend informed decision-making for all advanced CKD patients for their dialysis modality selection [6, 7]. This requires individual patients/caregivers to comprehend the complex medical, social, and financial aspects of their dialysis options and select the modality best suited to their life. Unfortunately, awareness of CKD and its management options is low among advanced CKD patients, which serves as a major limitation to greater home dialysis use [1, 8]. Several cohort and a few randomized studies, largely from outside the US have shown that comprehensive pre- kidney failure disease education (CPE) improves CKD awareness and increases informed home dialysis selection and use, with the reported home dialysis rates ranging from 35 to 85% among the CPE recipients [9]. Studies have further shown that provision of CPE improves patient awareness and is associated with beneficial impacts on several pre-, and post-kidney failure outcomes [10, 11]. In recent times, we and others have shown that provision of a formal protocol-based CPE, incorporated within the clinical care or as a stand-alone service leads to greater home dialysis selection (50–74%) and use (30–62%) even among the US advanced CKD patients [12, 13].

Nevertheless, provision of kidney disease education occurs uncommonly, in less than 1% of incident kidney failure requiring dialysis patients in the US, and its occurrence and practice patterns with VHA is not known [14]. More than half of these patients are recognized late in the course of CKD, and receive none to limited pre-kidney failure care from kidney disease specialist [1]. Even among those receiving longer nephrology care, lack of validated protocols, concerns regarding the selection bias in the available data, and lack of systemic models establishing feasibility limit a wider, more universal provision of CPE. The Trial to Evaluate and Assess the effects of CPE on Home dialysis in Veterans (TEACH-VET) is aimed to identify the burden of advanced CKD among Veterans through an EHR–based strategy, and assess the impact of universal CPE on the parameters of informed dialysis decision, home dialysis selection and use, and several related clinical, patient-centered, and health services outcomes.

## Methods

### Trial overview

**Fig. 1:**
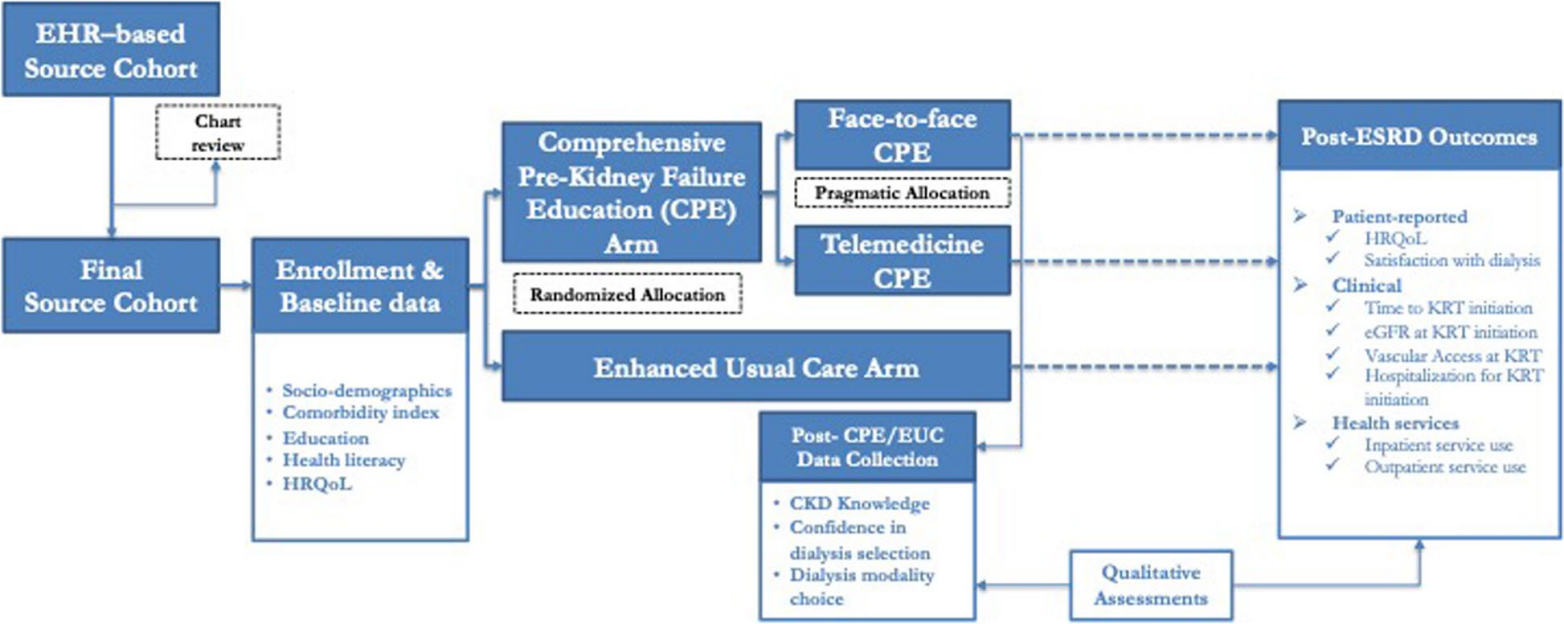
Overview of the Study Design and Study Activities for the TEACH-VET. EHR: electronic health records, HRQoL: health related quality of life, CPE: comprehensive pre-kidney failure education, EUC: Enhanced usual care, CKD: chronic kidney disease, eGFR: estimated glomerular filtration rate, KRT: Kidney replacement therapy

The overall design of TEACH-VET is depicted in Fig. 1. First, the study uses an electronic health record (EHR)–based strategy to identify veterans with advanced CKD (source cohort) and assess their status/need of ongoing care from kidney disease specialist and CPE. Then, in a multi-method randomized controlled trial (RCT), the study plans to enroll 544 Veterans from the source cohort, and randomize them in 1:1 allocation to the CPE/intervention arm vs. usual clinical care supplemented by kidney disease education material, enhanced usual care (EUC) arm. The study aims to compare the effects of intervention/control on parameters of informed dialysis decision and dialysis modality selection, dialysis modality use, and several clinical, patient-centered and health services outcomes post-kidney failure. The study also has a qualitative component, which uses semi-structured interviews to explore Veterans’ perceived satisfaction with CPE, their preferences for face-to-face or tele-CPE, and their perceived barriers and facilitators in the selection and use of preferred dialysis modality. The quantitative and qualitative data are collected and will be analyzed separately, and the results will be integrated for a more comprehensive understanding.

### Conceptual framework

The structure of TEACH-VET is based on the modified Bandura’s model of social cognitive theory [15]. Social cognitive theory identifies a set of core determinants including knowledge of health risks and health benefits from different health practices, self-efficacy, outcome expectations, and perceived facilitators and social and structural impediments to the changes they seek. These core determinants create the preconditions for, and play a central role in, human motivation, action, and health decisions. DeWalt et al. [16] successfully modified this model in a randomized evaluation of educational intervention in heart failure patients arguing that patient action, i.e., informed decision-making in TEACH-VET leads to positive changes in patient health-related outcomes.

### Hypothesis and rationale

**Fig. 2:**
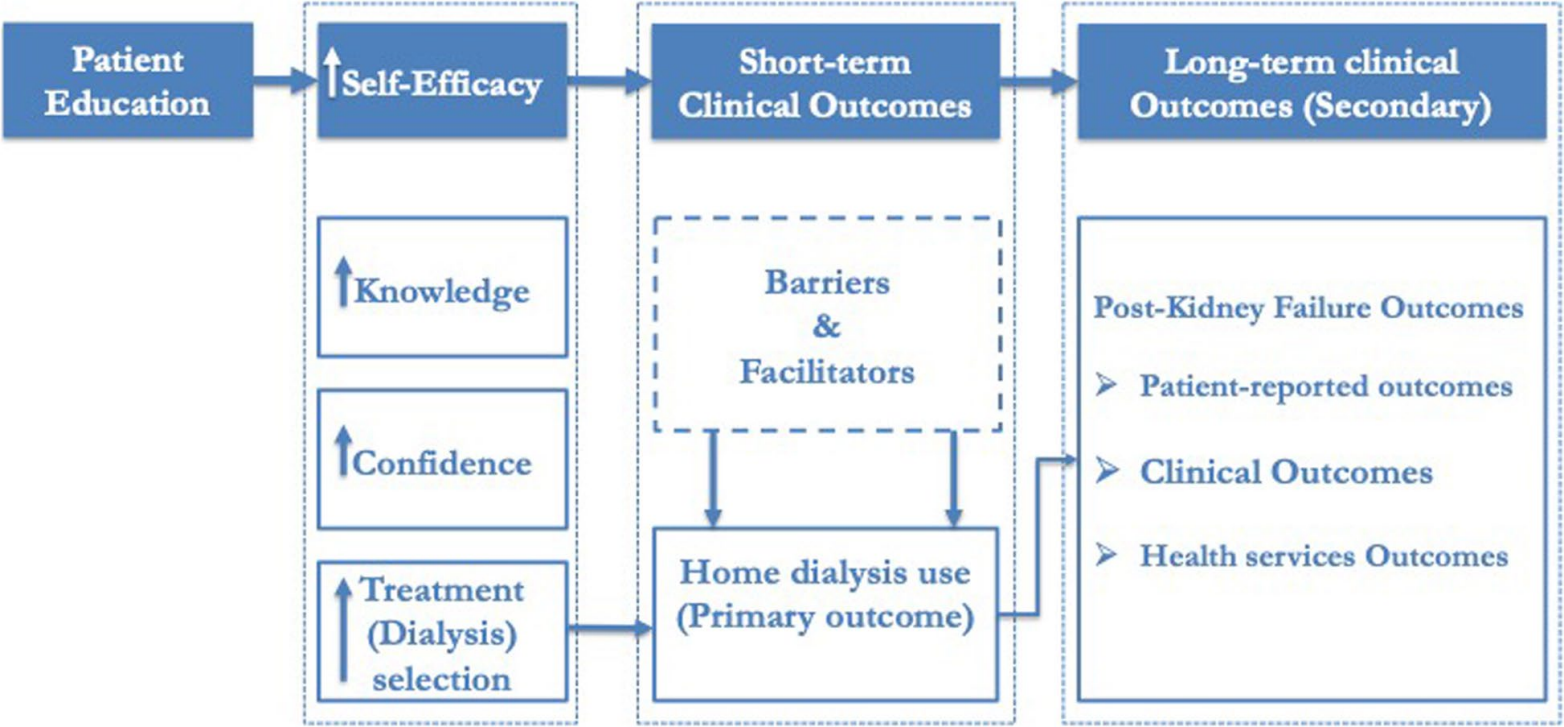
Conceptual Framework for TEACH-VET with Reference to the Study Outcomes

We hypothesize that a system-based application of universal CPE and patient-centered initiation of kidney replacement therapy (KRT) will increase home dialysis utilization and improve multiple clinical, patient-centered and health service outcomes (Fig. 2). Specifically, CPE will increase Veterans’ self-efficacy, i.e., knowledge of CKD and its management so that they become more confident in making an informed choice for their disease management and dialysis treatment. The contention is also that Veterans’ behavior post-CPE will lead to increased use of home dialysis compared to the usual care group (primary outcome). According to social cognitive theory, [16] individual person-level determinants (e.g., knowledge and confidence) may increase the likelihood of an individual’s executing a behavior (e.g., informed decision-making and self-management). Additionally, environmental factors can also influence behavior; as such, environmental factors the Veterans perceive as barriers and facilitators will be examined (qualitative phase). Finally, CPE-induced behavioral changes may show positive impact on post-kidney failure outcomes.

### Study population

TEACH-VET is approved by the University of Florida Institutional Research Board and registered with the clinicaltrials.gov (TEACH-VET, NCT04064086). All protocols involving human participants were in accordance to the institutional and national guidelines and in compliance with the Declaration of Helsinki. The study was launched in August 2020 across the North Florida/South Georgia (NF/SG) Veterans Health System (VHS), one of the largest VHS in the nation. Based on the recommendations by the professional nephrology organizations and CMS, TEACH-VET aims to target all adult (> 18 years old) Veterans with advanced stage 4 and 5 CKD not on dialysis for enrollment. The study excludes Veterans who are non-English-speaking, homeless or living in assisted living facilities, and with dementia or less than 6-months life-expectancy.

### Enrollment strategy

To ensure the enrollment targets all prevalent advanced CKD patients, in addition to directly approaching the Veterans attending the nephrology clinic, TEACH-VET recruits participants through our recently published, EHR–based ‘Opt-Out' Source Cohort Strategy [17]. In brief, a ‘source cohort’ of all actively registered Veterans at NF/SG VHS with ICD-10 codes for stage 4(N18.4) and 5 CKD(N18.5) or two latest outpatient estimated glomerular filtration rate (eGFR) of less than 30 ml/min at least 90-days apart is generated. The cohort is then sorted in a random order, and the potential participants are approached in consecutive order for their status/need for specialty nephrology care and CPE. All eligible and interested participants then undergo informed consent through a ‘waiver of documentation of informed consent’, approved by the University of Florida Institutional Review Board, and enrolled into the second phase RCT.

### Baseline data collection and randomization

Once enrolled, all participants provide baseline data comprising of patient-reported socio-demographics, education, household composition, and annual family income (Table 1). Participants are assessed for health literacy by Rapid Estimate of Adult Literacy in Medicine-short form, medical comorbidity by the Charleston Comorbidity Index, and HRQoL by Kidney Disease Quality of Life (KDQoL-36), excluding dialysis items [18–20]

**Table 1:** Key Variables and Quantitative Outcomes by the Time of Data Collection

Variable Domains and Outcomes	Variable Name & Characteristic	Time Point
Demographics (Baseline data)	Age Race/ethnicity Education Annual Family Income Social Support	Pre-Intervention Baseline
Comorbidities (Baseline data)	Comorbidity Index	Pre-Intervention Baseline
Health Literacy (Baseline data)	Health Literacy Score	Pre-Intervention Baseline
CKD/Kidney Failure Knowledge	CKD/Kidney Failure Knowledge	• Pre-Intervention Baseline • Post-Intervention/Control
Confidence in dialysis decision-making	Confidence in dialysis selection	• Pre-Intervention Baseline • Post-Intervention/Control
Dialysis Modality Selection	Dialysis modality selection	• Pre-Intervention Baseline • Post-Intervention/Control
Dialysis Modality Use **(Primary Outcome)**	Dialysis modality use	90-day post-kidney failure
Patient Reported	HRQoL	• Pre-Intervention Baseline • 90 days post-kidney failure
Patient Reported	Satisfaction with Dialysis	90-day post-kidney failure
Health Service Utilization	Number of inpatient stays Number of outpatient visits	Post-Intervention to 90-day post-kidney failure
Clinical	Time to KRT initiation eGFR at KRT initiation Inpatient initiation of KRT Vascular Access Presence at KRT Vascular Access Use at KRT	At the development of kidney failure

*KRT* Kidney Replacement Threapy

.

All enrolled participants are randomized by a computer-generated block randomization schedule devised by the study statistician, in 1:1 ratio into CPE or EUC arm. Considering the primary outcome of dialysis modality use and the strong known influence of socioeconomic factors, [21] the randomization is stratified by the stage of CKD (4 or 5) and annual family income (250% above or below federal poverty level adjusted for total number of household members) [22].

### Intervention/CPE arm

Participants and their preferred care partner(s) in the intervention arm receive a standardized, evidence-based, two-phase CPE by trained kidney disease educators in an Intent-to-Teach manner. The protocol covers the domains of education recommended by the professional nephrology organizations and CMS (Table 2), [4, 5, 23, 24] with an interactive, instructor-led audio-visual education, followed by individual patient-oriented counseling session that includes lifestyle simulation discussions. Prior studies have shown the advantages of such two-phase approach on comprehension, fears, and home dialysis selection [25, 26]. Over last decade, we have tested, refined and validated this protocol at two geographically distinct universities and affiliated VAs within the US to ensure literacy level and cultural relevance for the target patient population [12, 13]. For this study, we further pilot-tested the intervention with a local Veteran Engagement Committee made up of a diverse group of 12 Veterans and Veteran caregiver volunteers from Florida. This committee provided specific feedback to further hone the language used and explanations given for describing kidney disease and its management to fellow Veterans. Recently, we demonstrated our protocol can be delivered either face-to-face or through telemedicine with equivalent outcomes in terms of confidence in dialysis decision-making and home dialysis selection [26].

**Table 2:** TEACH-VET Comprehensive Pre-ESKD kidney disease Education (CPE) Protocol domains and Missions

Domains of the CPE	Missions/Messages of the CPE for Patients
• Location and Function of the Human Kidneys • Overview of Kidneys in Human Health o Excretory Functions of the Kidneys o Non-excretory Functions of the Kidneys ▪ Importance in cardiovascular health ▪ Importance in bone health ▪ Importance in Anemia • CKD and stages? o Differentiate CKD from Acute kidney injury • Understand Kidney Failure (ESKD) o Common Symptoms of Kidney Failure o Common Signs of Kidney Failure • Options for the management of Kidney Failure? o Kidney Transplantation o Conservative Care o Dialysis therapies ▪ Home-based Peritoneal Dialysis ▪ Home-based Hemodialysis ▪ Center-based Hemodialysis • Lifestyle on Dialysis • Frequently Asked Questions	• CPE should be available to all patients with stage 4 and 5 CKD, irrespective of their socio-demographic and comorbidity status, or perceived eligibility for home dialysis therapies • For eligible patients, kidney transplantation is the best modality of renal replacement therapy • It is important to know the cause of transplant ineligibility, and the possible corrective measures • All dialysis modalities have equivalent medical outcomes • Unless deemed medically/socially unsuitable by the provider, the choice of dialysis modality is a patient and caregiver’s decision and should be targeted as a shared decision-making process • Avoid fear as an overbearing motivator for dialysis modality selection by ensuring the patients that the routine care should provide adequate support for any of the modalities chosen for most patients • Decision for dialysis should be attempted early in the course of advanced CKD, if possible, by the end of the CPE session. If not feasible, the patient must plan to attend additional CPE sessions. • All patient selections should be evaluated for confidence in dialysis decision making, with the options for patients with low confidence to attend follow up sessions

*CPE* Comprehensive pre-ESRD education, *CKD* Chronic kidney disease, *ESKD* End stage kidney disease

To ensure the intervention is standardized and uniform throughout the study, the kidney disease educators are trained by licensed nephrology providers in the content, and by experienced patient educators in the delivery of the CPE prior to their involvement in the study. Additionally, with the participants’ permission, all CPE sessions are recorded for the first 3 months of the study or after initiation of the new educator, and 10% of randomly selected CPE are recorded throughout the study period. The recorded data is reviewed for credibility, competence, and thoroughness of the educator interactions during CPE. Finally, the study tracks the amount of time educators spend with each participant for individual counseling, reviews the fidelity of important pre-defined topics and their delivery, and keeps detailed notes of any deviations from the CPE protocol. Feedback and additional training is provided as needed to ensure uniformity and standardization. Patients having any question or concerns after education are provided the opportunity to discuss with a licensed dialysis nurse or provider proficient in all KRTs.

**Fig. 3:**
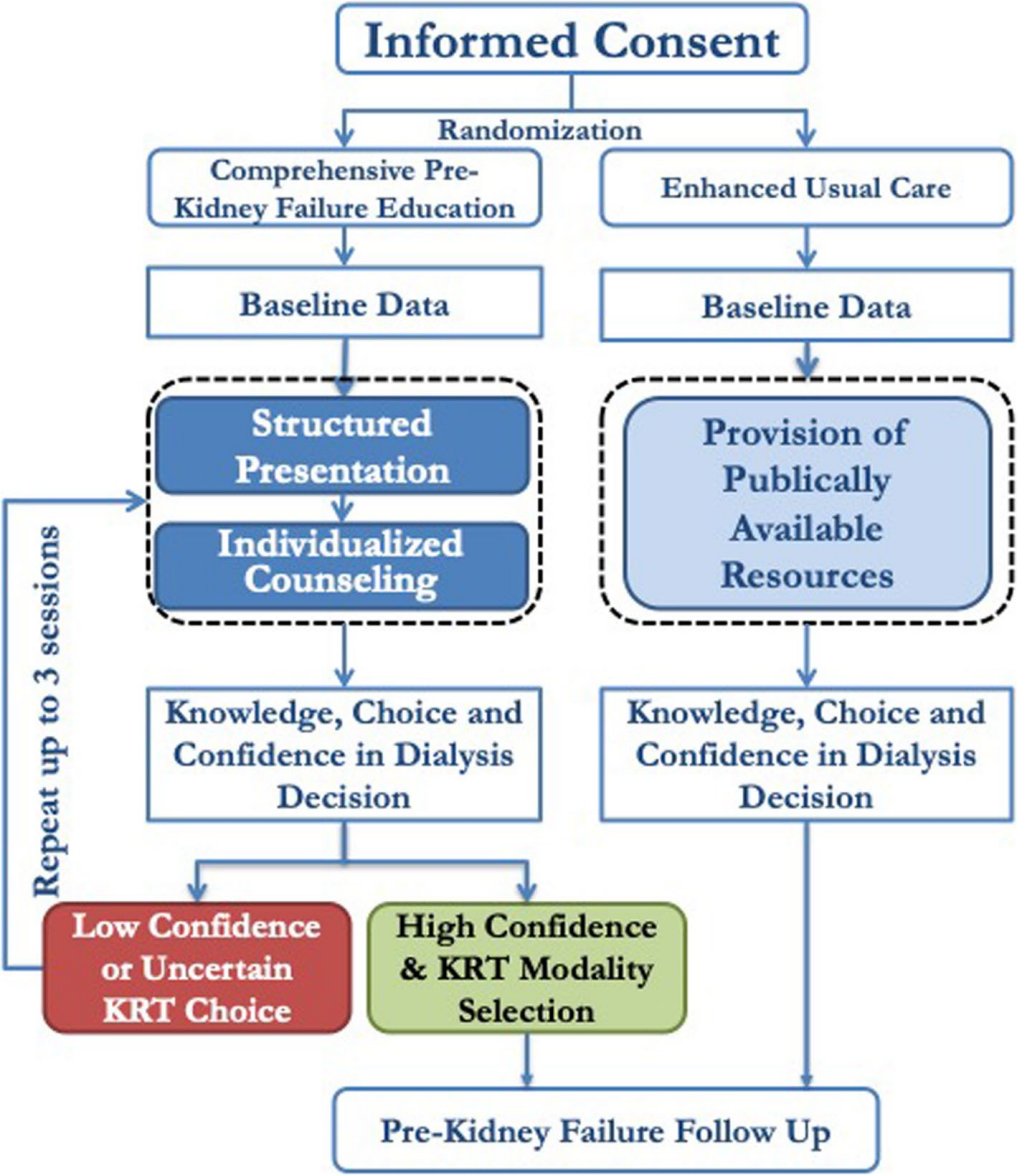
Intent-to-teach application of the Comprehensive Pre-kidney failure disease Education and Data Collection. KRT: Kidney Replacement Therapy

To ensure informed dialysis selection, participants are assessed for their confidence in dialysis decision-making and selection of dialysis modality at the end of the CPE session. Intent-to-Teach is assessed by confidence for dialysis decision making (defined by confidence rating of “quite confident” or “very confident”); those with suboptimal scores (“not at all confident” or “a little confident”) or “uncertain of the dialysis modality choice,” are advised to undergo repeat CPE sessions at an average of weekly intervals for a total of up to three counseling sessions (Fig. 3). Our pilot studies show a vast majority of CPE recipients reach an informed decision by 3 sessions; when optional, 84% prefer to attend only one session, and when mandated for clinical care or research 96–99% of the patients reach informed dialysis selection by 3 sessions [12]. Considering our preliminary data and to ensure the model is ready-for-dissemination, TEACH-VET allows CPE participants to pragmatically choose the method for CPE, either face-to-face, or through tele-medicine to the affiliated outpatient clinic or within their homes. We will analyze the differences in outcomes between these delivery methods in our secondary analyses.

### Control/EUC arm

Participants in the EUC arm are provided printed hand-outs directing them to online self-learning CKD resources, freely available through several professional nephrology organizations, including the VA [4, 5, 23, 24].. While the investigators acknowledge the scientific need for an unaltered control arm, enhancing ‘usual care’ through provision of the self-learning resources was considered the appropriate ethical compromise. To mirror the expected duration between the pre-, and post-CPE data collection in CPE arm, EUC arm participants provide data for post-EUC knowledge, confidence in dialysis decision-making, and dialysis modality selection 10-days after the provision of the self-learning resources.

### Qualitative study

The qualitative study employs a maximum variation sampling strategy to ensure a diversity of demographic and clinical characteristics [27]. Fifteen Veterans from each of the face-to-face-CPE, tele-CPE and EUC groups respectively are interviewed by telephone for 45–60 min using a semi-structured interview guide based on the Theoretical Domains Framework (TDF) [28, 29]. Furthermore, an additional 15 Veterans who did not ultimately use their preferred dialysis modality are interviewed 90-days post-kidney failure to explore experiences and barriers. The TDF supplies the working analytical framework for identifying factors that influence Veterans’ informed dialysis decision-making and experience with different dialysis modalities, including any perceived factors influencing dialysis decision-making, perceived barriers to home dialysis selection and use, and [for CPE arm] satisfaction with education session and counseling. The verbatim transcriptions for the audio-recorded interviews will be analyzed by two independent coders, organizing the data by domains of the framework, e.g. Knowledge: participants’ knowledge regarding dialysis; Beliefs about capabilities: participants level of confidence; Intentions: CKD management preferences; Social influences: influence of family members, friends, or caregivers; Beliefs about consequences: expectations about CKD management and evaluation of results; Optimism motivation to recommend dialysis to other patients; and Emotions: feelings about CKD treatment options.

### Post-CPE/EUC follow up

Nephrology, and if not available, the primary providers for the participants are informed of the participants’ preferences for dialysis modality, with a plan for finalization of modality through clinical interactions. This communique further instructs the providers regarding the need and importance of the pre-kidney failure nephrology care, and the processes and desired timings for the peritoneal dialysis catheter insertion/vascular access creation. The providers are also informed about need to appraise study team with any concerns with the planned dialysis modality and the contact information and approval processes for the VA-facilitated KRT services. Participants are then followed by EHR reviews at quarterly and by telephonic interviews at semi-annual intervals to assess their need/status of dialysis therapies and any changes in their preferred dialysis modality. Participants in CPE arm are allowed to re-access the audio-visual group education session independently throughout the study period. All outcome measures and their collections timings are available in Table 1.

### Outcome measures

Study outcomes and their time of collection have been detailed in Table 1. In brief, CKD awareness is assessed by the prior validated instrument by Wright et al. [30] Considering this and other similar validated CKD knowledge instruments lack the domains of kidney failure or KRT knowledge—essential for informed dialysis decision—the team has developed and pilot tested a 29-item KRT knowledge questionnaire [26]. This questionnaire will be further refined during the TEACH-VET, and the team will report on its findings. Confidence in Dialysis Modality selection is assessed by a single item scale rated as “not at all confident” (0–20%), “a little confident” (20–40%), “somewhat confident” (40–60%), “quite confident,” (60–80%), and “very confident” (80–100%). Dialysis Modality Selection is documented for all patients with a single question: “If I had to choose a dialysis modality option today, I would choose (a) peritoneal dialysis; (b) home hemodialysis; (c) in-center hemodialysis; (d) conservative care; (e) I do not know”, with peritoneal dialysis and home hemodialysis aggregated as home dialysis.

TEACH-VET uses structured surveys conducted at the day-90 after starting dialysis for its post-ESRD clinical, patient-centered and health services outcomes, including the primary outcome of home dialysis modality use. This will allow period for transition to home dialysis among those requiring emergency initiation of dialysis or longer time for VA approvals. At the same time, allowing 90-day on dialysis will avoid erroneous over-ascertainment of those with primary home dialysis failure as long-term home dialysis users. Satisfaction with dialysis modality is assessed with a one-item satisfaction survey developed and validated by Juergensen et al. [31]

### Statistical considerations

We used G*Power version 3.1.9.2 for sample size calculations, which are based on the primary outcome of (home) dialysis use. Using one-tailed test with alpha of 0.05 and 80% power, to detect doubling of home dialysis use in CPE relative to EUC arm —estimating home dialysis actual use to be 10% for EUC (based on the prevalent data) and 20% for CPE—yielded a total sample size of *N* = 108 (54 per arm). Allowing the potential of attrition and missing data that cannot be accommodated by the proposed missing data handling techniques (up to 20% data loss), we will need 136 (68 per arm) to reach kidney failure requiring dialysis to allow detection of this clinically meaningful effect size. Considering we expect about 25% of the study participants to reach kidney failure through the study period, we plan to enroll 544 Veterans with advanced CKD for the study.

### Analytic plan

We will use multiple regression analysis to examine the effect of the CPE intervention on Veterans’ knowledge of CKD and confidence in dialysis decision making post- intervention or EUC. We will include the baseline knowledge and confidence scores as covariates in the model, to account for pre- intervention/EUC values. We will use orthogonal Helmert contrast codes to test for the effect of both CPE as a whole (collapsed across telehealth and face-to-face delivery methods) vs EUC, and for the effect of tele-CPE vs face-to-face CPE. (Although we do not predict an effect of treatment delivery method, we have planned to include the comparisons derived by the Helmert contrast coding to test and account for any variance that may be introduced by different treatment delivery methods, should such variance/effect emerge.) We will use multiple logistic regression to examine the effect of CPE on Veterans’ initial selection of home dialysis; specification of this logistic regression model for home dialysis initial selection mirrors the regression models for confidence and knowledge, with the exception that the outcome is binary. Additionally, we will use logistic regression with Helmert contrast coding to compare home dialysis actual use between CPE and EUC groups, as well as between tele-CPE and face-to-face-CPE groups (within the overall CPE group). This multiple logistic regression for actual use of home dialysis constitutes the analysis for the primary outcome of this study.

For continuous secondary outcomes post-kidney failure (e.g., HRQoL), we will use multiple regression analysis with Helmert contrast coding for CPE and EUC comparisons (as used in above regression models), an effect of dialysis modality actually used (home dialysis vs in-center dialysis), and interaction effects between the contrast codes and dialysis modality ([CPE-vs-EUC*Modality] and [tele-CPE vs face-to-face CPE*Modality]. Where applicable, we will include the outcome’s baseline scores and/or other relevant covariates. For dichotomous secondary outcomes post-kidney failure (e.g., inpatient initiation of dialysis), we will use multiple logistic regression analysis, with the specification of this model mirroring that for continuous secondary outcomes, with the exception that the outcome is binary. Finally, for the secondary outcome of time to kidney failure, we will calculate a Kaplan-Meier estimate.

For qualitative sub-study, TDF will supply the working analytical framework [28]. Two researchers will independently code first few transcripts using the framework, reading transcripts line-by-line to capture as many behaviors, values, emotions, and impressions as possible, and comparing results to ensure everything relevant was coded according to the constructs of the framework. An iterative process will be used to refine themes from the framework based on patterns in the data, generating a thematic map [32]. The qualitative data analysis and interpretation will be integrated into the quantitative findings to provide in-depth understanding of the barriers Veterans experience in acquiring the knowledge needed to manage CKD, and facilitators involved in their selection and use of a patient-centered KRT.

## Discussion

The burden of progressive CKD transitioning to kidney failure requiring dialysis is large for patients and healthcare system, and there are several critical systemic deficits in the care of these patients in the current infrastructure. Among these, lack of opportunities for informed dialysis selection and gross underuse of home dialysis have been important, long-targeted yet underachieved concerns. Available studies show that providing CPE can substantially improve these concerns at institutional levels, however, we lack randomized studies, validated protocols, and implementation models to address these concerns at a systemic level. TEACH-VET attempts to examine and address several of these concerns.

Nearly half of incident kidney failure patients requiring dialysis have none to limited (less than 6-months) pre-kidney failure nephrology care.^1^ These patients have low probabilities for acquiring specialty care or CPE necessary to reach informed dialysis selection, and thus, home dialysis use. Studies have shown EHR-based screening is accurate to a sensitivity and specificity of 99% for the identification of stage 3 or higher CKD. However, these models are not routinely used to identify and improve clinical care in advanced CKD [33]. TEACH-VET will aim to identify all Veterans with advanced CKD within the VA database through an EHR-based source cohort, and evaluate their status/need for specialty nephrology care and CPE. If validated, this will provide a blueprint for developing such models in similar mid-large healthcare infrastructures across the country.

Several cohorts and a few randomized studies from outside the US have shown CPE increases informed home dialysis use. Over last decade, a few cohort studies from within the US have validated these findings [9]. Unfortunately, interpretation these results is limited by the concerns for selection bias. Furthermore, in the only available randomized study examining the impact of patient education strategy on home dialysis in the North America, Canadian investigators only evaluated the patient selection, and not use of home dialysis. Randomized evaluation of universal CPE for all advanced CKD patients has not been tested till date in the US general or Veteran populations. The results of TEACH-VET will provide evidence to universalize CPE across the sociodemographic and comorbidity spectrum, and identify limitations related to this strategy. Furthermore, assessment of the parameters of informed decision making, i.e., improvement in CKD/KRT knowledge and confidence in dialysis decision-making will further assist in differentiating between a patient-centered vs. system-driven increase in home dialysis use.

Lack of validated protocols hamper wide-spread adoption of CPE in routine clinical practice. Several private and public organizations, including VA, have recently launched technology-based solutions pooling resources and expertise to a central organization with capacity to reach patient-base beyond individual practices [34]. The effects of such programs have been limited and ill-quantified to date. We have developed and tested our easy-to-implement CPE protocol in different clinical models, i.e., incorporated within the clinical care, as a stand-alone model, and through telemedicine-based delivery. TEACH-VET integrates this further at a systemic level with both face-to-face and telemedicine-based delivery, and assesses their effects on parameters of informed decision-making, with appropriate measures to avoid physician-driven modality selection. The qualitative component further assesses the patient-preferences for such services and their barriers. These results will provide the necessary evidence to use telemedicine technology for wider dissemination of these services.

The cost-effectiveness of CPE and home dialysis have been demonstrated in health economics models. Despite these, need for significant resources, including trained specialists capable of providing CPE have limited routine provision of CPE in clinical practice at systemic levels [14]. Prospective randomized assessments of the inpatient and outpatient service utilizations will provide guidance to the health services outcomes in the care of advanced CKD for a universal system-based approach.

While studies have assessed the effects of CPE on home dialysis selection and use in general population, the data on Veterans are limited. Veteran kidney failure population requiring dialysis is known to be significantly older and with greater functional limitations. Furthermore, most Veterans (about 90%) receive their CKD care from within the VHA but, only a minority (about 10%) receive their KRT from the VHA. This disconnect hinders CPE and planned transition to KRT and resultantly, the home dialysis utilization. Home dialysis rates among Veterans with kidney failure requiring dialysis (about 7%) are significantly lower than already low rates prevalent in the US general population [1, 35]. TEACH-VET will evaluate a system-based approach in an area of unmet need and systemic deficit in the care of Veterans with advanced CKD. Finally, the qualitative assessments of Veterans status/preferences for receiving the specialty nephrology care, CPE, and home dialysis therapy have not been performed to date. Together, the results will provide targeted Veteran-specific data, instrumental for future research, while establishing a ready-to-implement model for dissemination across the VHA system.

Several cohort-based studies have shown the benefits of CPE on a variety of pre-, and post-kidney failure outcomes, including quality of CKD care, time to kidney failure, vascular access outcomes, and pre- and post-kidney failure survivals [10, 11, 36]. While not powered to detect differences in these outcomes, TEACH-VET will assess a variety of clinical, health services and patient-centered outcomes once these patients develop kidney failure requiring dialysis.

There are few limitations of TEACH-VET. While the study investigates the status/need of the pre- kidney failure nephrology care and empowers informed dialysis selection, it doesn’t mandate protocol-based congruence for new nephrology referrals or provider adherence to patient-selected dialysis modality. Thus, by design, it assesses the effects of a stand-alone CPE program superimposed on routine nephrology care. The study will report the effects of intervention/control on these events, and the pre-planned subgroup analyses and qualitative assessments will evaluate the impacts of such uncontrolled variables on the study outcomes. Second, to ensure the need for emergent dialysis due to patients’ comorbidities or administrative limitations of infrastructures providing nephrology care within and outside VHA do not impact evaluation of long-term dialysis modality use, TEACH-VET will assess the home dialysis use at 90-day post-kidney failure. The study will report these occurrences, and document any difference between the chosen vs. initial modality, and the qualitative assessments will attempt to dissect the facilitators and barriers to their initiation of chosen modality for eventual systems improvements. Finally, we acknowledge the results of TEACH-VET will only provide evidence for adopting and disseminating these strategies within the unique healthcare infrastructure of VHA. Adoption of the findings in the general US healthcare system will require additional studies establishing its efficacy and feasibility.

To summarize, studies from around the world as well as from within the US have shown that comprehensive pre-kidney failure education may have substantial benefits in the clinical care of advanced CKD, but the evidence to support this effectiveness has not been obtained from well designed, randomized controlled studied from within the US. Furthermore, we lack validated protocols and feasible systemic models to deliver CPE. TEACH-VET aims addresses these deficits through a system-based approach for universal CPE within VHA, delivered via either an in-person visit or telemedicine and investigates its impact on Veterans’ informed dialysis choice and home dialysis rates. Findings from this study will demonstrate whether such a universal approach can improve Veterans clinical, patient-centered and health services outcomes. If successful, this will provide evidence for policymakers to expand and implement such programs across the healthcare system to improve care for patients with advanced CKD, increase home dialysis use, and improve post-kidney failure outcomes, while reducing health service utilization and cost.

## Data Availability

Not applicable.

## Abbreviations

CKD: Chronic Kidney Disease
CPE: Comprehensive pre-kidney failure disease education (CPE)
EHR: Electronic health records
ESKD: End stage kidney disease
KRT: Kidney Replacement Therapy
TEACH-VET: Trial to Evaluate and Assess the effects of CPE on Home dialysis in Veterans
RCT: Randomized Controlled Trial
VHA: Veterans Health Administration
